# Supplementary figures and images for: Semantics of Dairy Fermented Foods: A Microbiologist’s Perspective
Source: Foods. 2022 Jun 29;11(13):1939. doi: 10.3390/foods11131939 (PMC9265904; doi:10.3390/foods11131939)

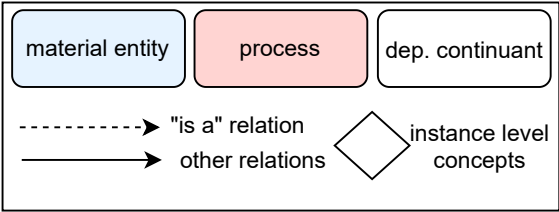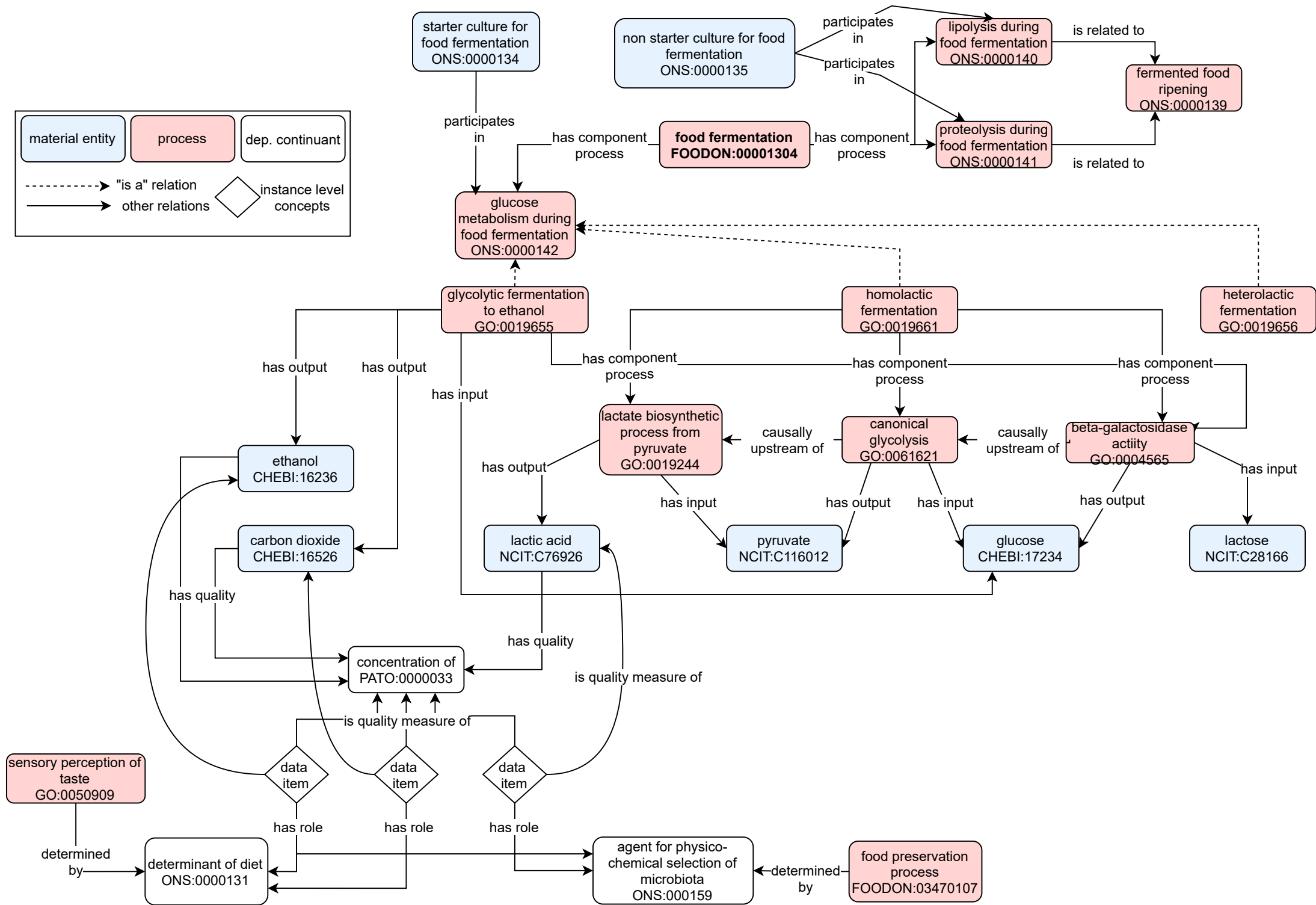

Supplement: Supplementary file 1 [file foods-11-01939-s001.zip › foods-1756281-sm.pdf]
